# Central recirculation zone induced by the DBD plasma actuation

**DOI:** 10.1038/s41598-020-70116-9

**Published:** 2020-08-03

**Authors:** Gang Li, Xi Jiang, Zhijun Lei, Cunxi Liu, Jinhu Yang, Yanji Xu, Gang Xu

**Affiliations:** 1 Key Laboratory of Light Duty Gas Turbine, Institute of Engineering Thermophysics, Chinese Academy of Sciences, Beijing, China; 2 University of Chinese Academy of Sciences, Beijing, 100049 China; 3 School of Engineering and Materials Science, Queen Mary University of London, Mile End Road, London, E1 4NS UK

**Keywords:** Energy science and technology, Engineering, Physics

## Abstract

Central recirculation zone (CRZ) is commonly formed in the near field of the injector exit by the vane swirler and used to stabilize the flame. In our experiment, a CRZ induced by the DBD plasma actuation was observed in the low swirl burner configuration for the first time, which clearly demonstrated that the mechanism of the combustion control by the plasma swirler is mainly through the aerodynamic effect. Three dielectric barrier discharge (DBD) actuators are placed in a circular array around the axis to generate ionic wind in the circumferential direction of the injector. Characteristics of the flow field have been measured using Laser Doppler Anemometry. It is found that a central recirculation zone with the shape of an ellipsoid is formed in the non-reacting flow field with the plasma actuation. The position of the upstream stagnation point was determined by the strength of the actuation. Although the CRZ disappears in the reacting flow field as the result of combustion heat release, the influence of the discharge on the flame lift-off height is noticeable. The results demonstrate that swirl enhancement by the plasma swirler is feasible, flexible and effective as a non-intrusive measure for flow control.

## Introduction

The history of application of thermally-equilibrium plasma or thermal plasma for initiation of combustion started over a century ago^1,2^. Plasma based ignition devices for practical applications such as internal combustion engines have attracted continuous efforts, e.g.^3^. In recent decades, particular interest appears in non-equilibrium plasma, which consumes a small amount of energy, for ignition and combustion control^4^. In aerodynamics, flow active control using non-equilibrium or low-temperature plasma is an area of increasing interests^5,6^. Plasma may have different physicochemical effects on flow and combustion processes, depending on the type of plasma, which can produce not only heat, but also electrons, ions, long-lifetime intermediate species, radicals, and excited molecules. With the presence of plasma, ionic wind, large density gradient, and Coulomb and Lorentz force can affect flow and combustion via different ways^7^, such as generating force (aerodynamic effect), changing the transport properties, changing the kinetic pathways, as well as the thermal effects. Accordingly, a wide range of flow and combustion control applications can be found, e.g. promising applications such as controlling laminar-to-turbulent transition in boundary layers, flow instability, ignition and flammability range in combustion applications^8^. For laminar-to-turbulent transition, it was identified that a 15 cm long plasma gun was effective in delaying the transition^9^, where the plasma actuation increased the length of laminar flow from 3 to 10 cm. The turbulence evolution of a plasma jet powered by a nanosecond generator was observed using the Schlieren technique coupled with a high-speed imaging camera^10^, where it was found that flow instabilities were correlated with the high voltage pulses.

Plasmas can be divided into two categories, i.e. thermal plasma (or equilibrium plasma) and non-equilibrium plasma. They are quite different in their characteristics. For thermal plasma, there is an equilibrium between the electronic, rotational and vibrational temperatures of particles, and the neutral gas temperature and electron number density are very high^7^. When this type of plasma is present in the flow and combustion fields, the observed effects are mainly thermal and the plasma energy consumption is relatively high. Among different types of plasmas, spark and arc discharges are close to equilibrium plasmas, with a high neutral gas temperature and electron number density but a low electron temperature and consuming a large amount of energy. Thermal plasma has found many applications in combustion including ignition, while the recent applications are increasingly on non-equilibrium plasma. For non-equilibrium plasma in which the electronic, rotational and vibrational temperatures are very different, the neutral gas temperature and electron number density are relatively low, but it has higher electron temperature (1–100 eV) and is more kinetically active due to the rapid production of active radicals and excited species^7^. The effects on flow and combustion by non-equilibrium plasma strongly depend on plasma properties, i.e., the electron temperature and electron number density, which are governed by the reduced electric field (the electric field strength divided by the molecular number density) and can be controlled to a certain degree. One of the commonly used non-equilibrium plasmas is the dielectric barrier discharge (DBD) plasma, where the average energy of electrons can be modified by changing either the gas pressure (or gas density) or the width of discharge gap. Thereby non-equilibrium plasma offers new opportunities in flow and combustion control.

The physicochemical effects of DBD plasmas have led to a number of flow control applications of plasma actuation, including aerodynamic applications such as drag reduction, lift enhancement, boundary layer separation/transition control, and noise reduction, as well as mixing enhancement in flow and combustion applications^6,11–13^. Plasma actuators are generally robust, light weight and small size, in addition to other advantages such as no moving parts and drag penalty, quick response, easy installation and low power consumption^11–13^. Traditionally, the DBD plasma actuations have been used to suppress flow separation for aerodynamic drag reduction in two ways. One way is placing the actuators just upstream of the separation point to add momentum to the boundary layer. Another way is triggering the transition of laminar flow to turbulent flow to withstand flow separation^11–13^. Meanwhile, swirling flow is widely used in premixed combustion to enhance flame stability through the induced central recirculation zone (CRZ) or the central velocity decay. The former is the feature of high swirl injector (HSI) and the CRZ provides a stable heat source for continuous ignition of the fresh reactants^14–16^. The latter is the feature of low swirl injector (LSI) where the central velocity decay induced by flow divergence keeps the lifted flame stable^17^. The concept of LSI was developed more than two decades ago^18,19^. The lifted flame can prevent the flame from flashing back and overheating the burner, which is particularly important for the combustion of fast burning fuels such as hydrogen-enriched fuels^20^. The associated flow field and emission characteristics have been reported for various gaseous fuels, including natural gas, hydrogen, syngas and liquid fuels^21–23^. Reacting flow field of the LSI is characterized by the absence of the CRZ which helps to reduce NOx production by shortening the reactants residence time^24,25^. The non-reacting flow field of the LSI is relatively uniform, without or with a suspended weak CRZ.

Flame stability under lean burn conditions is becoming increasingly important because of the needs to mitigate combustion pollutant emissions. Previous experiments show that the plasma swirler is effective in controlling the non-swirling jet flow and flame^26,27^ and the lift-off height of the flame^28,29^, but its mechanism is not very clear. Effects of active particles, heat release and aerodynamics are coupled together in the plasma combustion control. Which effect is dominant still needs to be further clarified. In this study, a CRZ induced by the DBD plasma actuation was observed in our burner configuration for the first time, which clearly demonstrated that the mechanism of the combustion control by the plasma swirler is mainly through the aerodynamic effect.

The LSI was chosen in the study, because the lift-off height is a critical parameter which influences the flame stability and thermal load of the injector. The lifted flame protects the actuators from being burnt-out, in addition to other advantages of the combined vane-plasma swirler^29^. In the experiments, the effects of the plasma actuation can be distinguished through the changes of the lift-off height, flow divergence angle and axial velocity decay rate. The plasma swirler was placed at the exit section of a LSI to adjust the swirl by changing the excitation voltage. Flame images were captured by a Nikon D3200 camera with f/5.6, ISO-3200 and exposure time of 1/40 s. Velocity distributions were measured by Laser Doppler Anemometry (LDA). Although plasma actuators were used in many previous applications, a central recirculation zone induced by the DBD discharges in a burner incorporating LSI has not been reported.

## Experimental method

**Figure 1:**
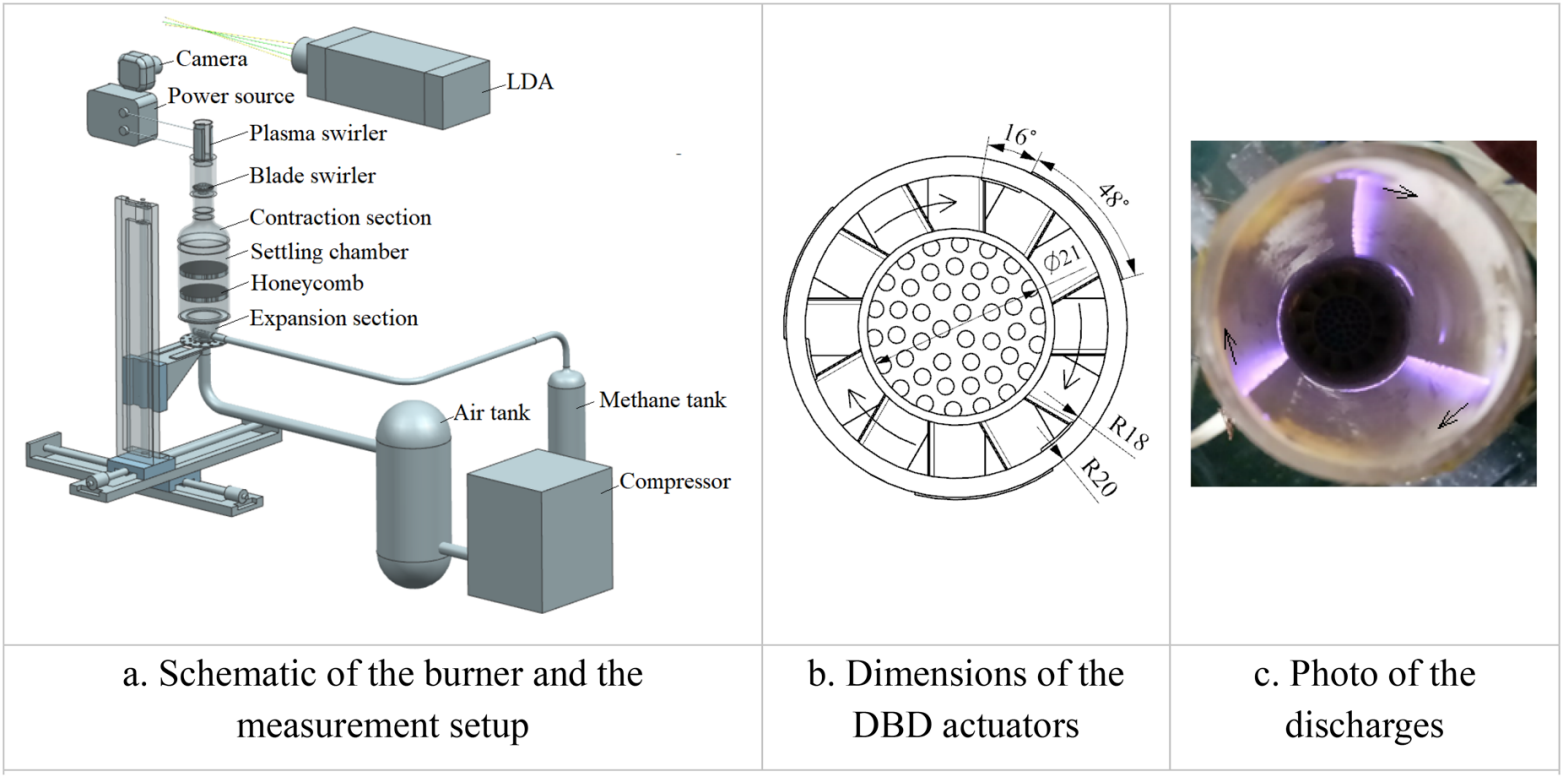
Schematic of the burner, the measurement setup, dimensions of the DBD actuators and a photo of the discharges.

The schematic of the experimental setup is shown in Fig. 1. The same experimental setup as that in our previous studies^28,29^ has been used. Air is supplied by a screw air compressor then stabilized in two tanks of 1.2 cubic meter. Mass flowmeters are used to regulate and measure the flow rate. Methane and air are mixed in the expansion section at the bottom of the burner before entering the settling chamber. Two layers of honeycomb in the settling chamber are used to remove the radial and circumferential velocity components. A contraction with an area ratio of 7.7:1 is connected to the settling chamber to further reduce the turbulence level. The flow field is measured by a two-dimensional LDA system with a measurement error less than 1%. The low swirl burner can be moved step by step in three directions with precision higher than 10 μm. The flame images are captured by a camera with exposure time of 1/40 s.

A plasma swirler installed at the exit section of the LSI is utilized to adjust the swirl. It consists of three DBD actuators in a circular array around the axis of the injector as shown in Fig. 1. The exit section of the injector is a quartz tube with a thickness of 2 mm and an inner radius of 18 mm. Electrodes of each actuator are separated by the injector. The electrodes on the inner side of the injector are activated by the plasma generator with width of 5 mm corresponding to 16˚ in the circumferential direction. The output waveform of the plasma generator is a sinusoid with a frequency of 9 kHz. The discharge waveforms acquired by Tectronic probes were presented previously^26^. The power consumptions of the discharge are about 16 W and 32 W for the applied voltage of 15 kV and 18 kV, respectively. The grounding electrode is three times wider than the activated electrode on the outer side of the quartz tube. The diameter of the central channel is 21 mm with a 135 mm recess length. The vane angle of the vane swirler is 34˚. The blockage ratio of the perforated plate is 0.6. A photo of discharges with 18 kV applied voltage is shown in Fig. 1. It can be seen that the plasma is distributed in a small area on the inner side of the injector. The distance between the rim of the injector and one end of the plasma actuators is 9 mm. The downstream flow of the vane swirler rotates clockwise as shown in Fig. 1. The direction of the plasma induced flow is the same as that of the main flow rotation, marked by the arrows in Fig. 1.

**Figure 2:**
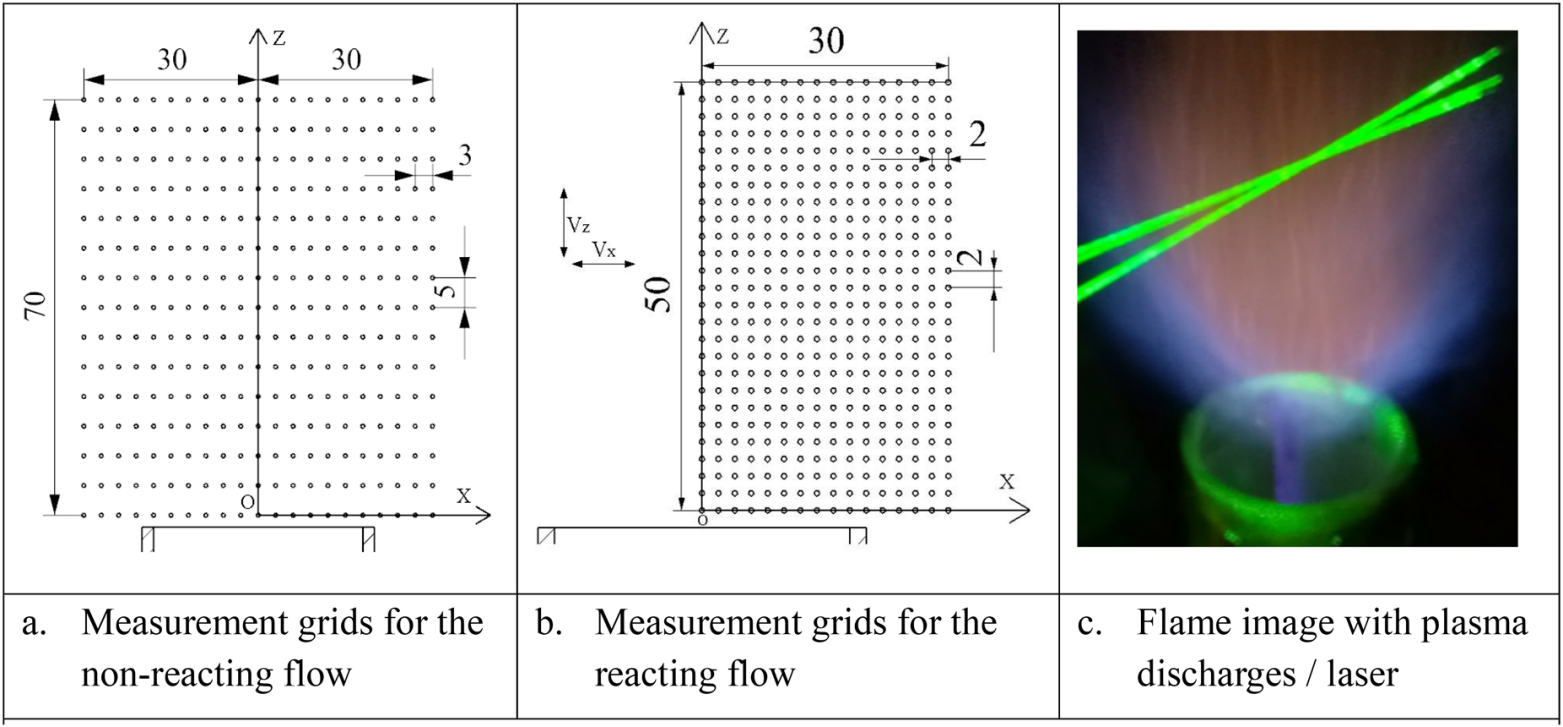
Measurement grids in the XZ plane and the flame image.

In the measurement, Cartesian coordinates are employed as the measuring frame. Figure 2a and b depict the measurement grids of LDA in the XZ plane for the non-reacting and reacting flows respectively. The flow fields are investigated in a 60 mm × 70 mm (non-reacting flow) and a 30 mm × 50 mm (reacting flow) rectangular regions, for the measurement grid of LDA in the XZ plane with Z representing the streamwise (V_z_ for the axial velocity component) direction and X the cross-stream direction (V_x_ for the radial velocity component). The origin point is 2 mm above the burner exit to avoid laser reflection from the rim of the injector. In the non-reacting flow measurement, the measuring points are 5 mm apart vertically in the stream direction Z and 3 mm apart in the cross-stream X direction. A uniform grid interval of 2 mm is adopted in the reacting flow measurement.

Figure 2c shows an image of the flame with the discharges and the laser. The optical path of a two-dimensional LDA is carefully calibrated to ensure adequate data collection rate. The LDA was manufactured by TSI equipped with two diode pumped solid state laser (300 mW each). The time-averaged velocity and its fluctuation were calculated based on nearly 5,000 samples at each grid within a 10 s sampling time. The yellow color in the center of the flame is caused by the tracing particles of TiO_2_.

## Experimental results

The flow rate for the CH_4_/air mixture is 150/12 L/min, which corresponds to the injector bulk flow velocity of 2.7 m/s. The equivalence ratio is 0.76 and the Reynolds number is about 6,335 based on the injector diameter. High Reynolds number flow is considered in this laboratory study for the low swirl injector, because the bulk flow velocity should be relatively high to match the flame propagation speed, in order to ensure the stability of the flame. In addition, most of the practical applications of combustion are in the turbulent flow regime. In the non-reacting flow field measurement, air was used in place of air/CH_4_ in the reacting case with the same volumetric flow which means the air volumetric rate is 162 L/min.

### Non-reacting flow field measured by LDA

**Figure 3:**
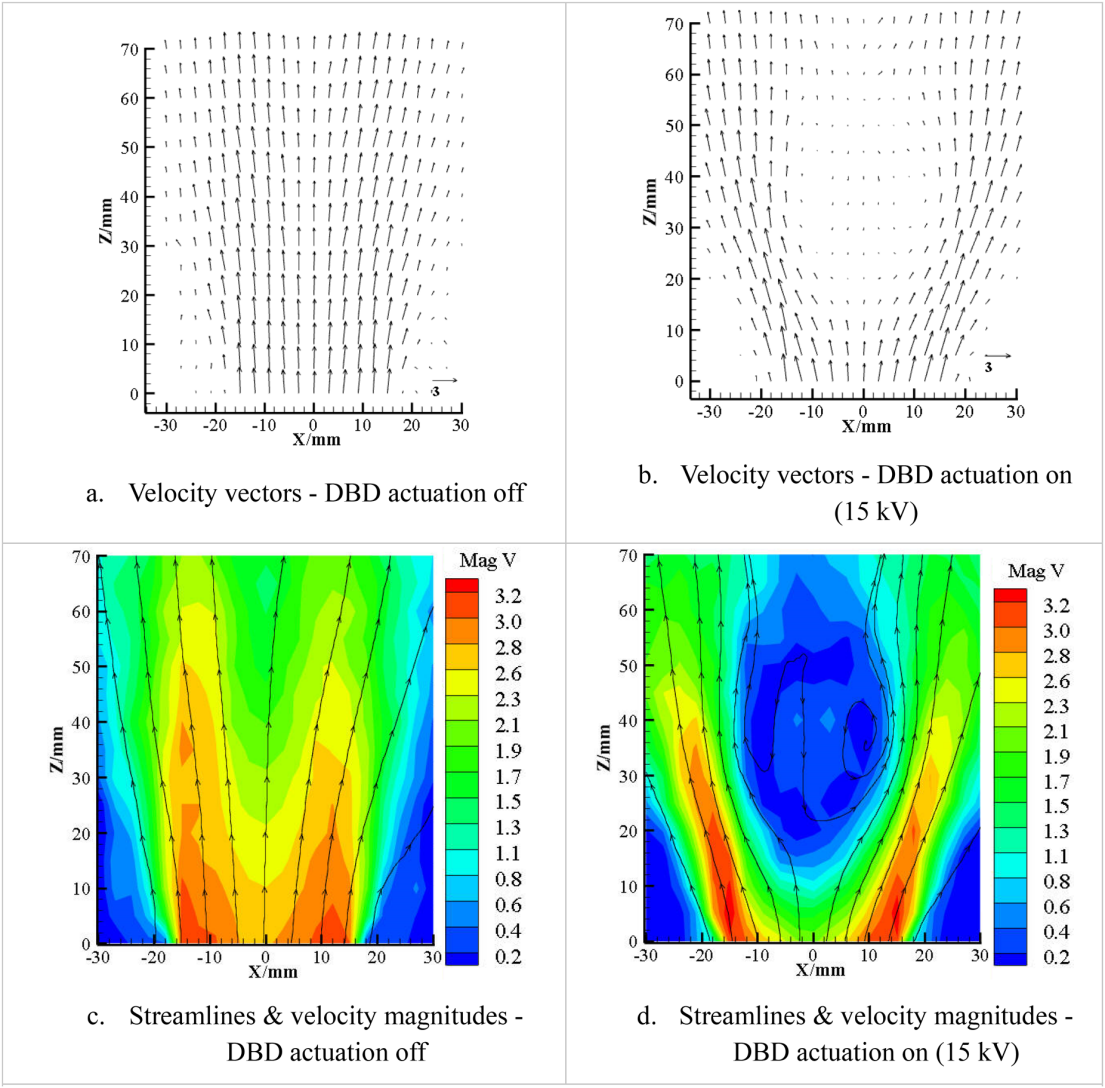
Non-reacting streamlines and velocity magnitude contours obtained by LDA (m/s) with the air volumetric rate at 162 L/min in the XZ plane.

Figure 3 shows velocity vectors, streamlines and contours of the velocity magnitude distributions of the non-reacting flow in the XZ plane. With the actuation switched off, the flow exhibits typical features of low swirl flow with flow divergence and central velocity decay as shown in Fig. 3a and c. The main function of the weak swirl is to induce radial mean pressure gradients leading to flow divergence but not recirculation^18^, while varying swirl changes the degree of divergence. With the actuation on, a CRZ is formed as shown in Fig. 3b and d. The CRZ is approximately axisymmetric and suspended in the flow like a bubble. It has the shape of an ellipsoid and its axial size is almost twice of its radial size. The results show that the swirl as well as the axial adverse pressure gradient are increased because of the plasma actuation and the induced reverse flow is sufficiently strong to result in a CRZ, but the increase is not big enough to cling the CRZ to the rim of the injector. Due to the blockage effect of the CRZ, the velocity in the jet shear layer and the flow divergence increase with the presence of plasma excitation.

**Figure 4:**
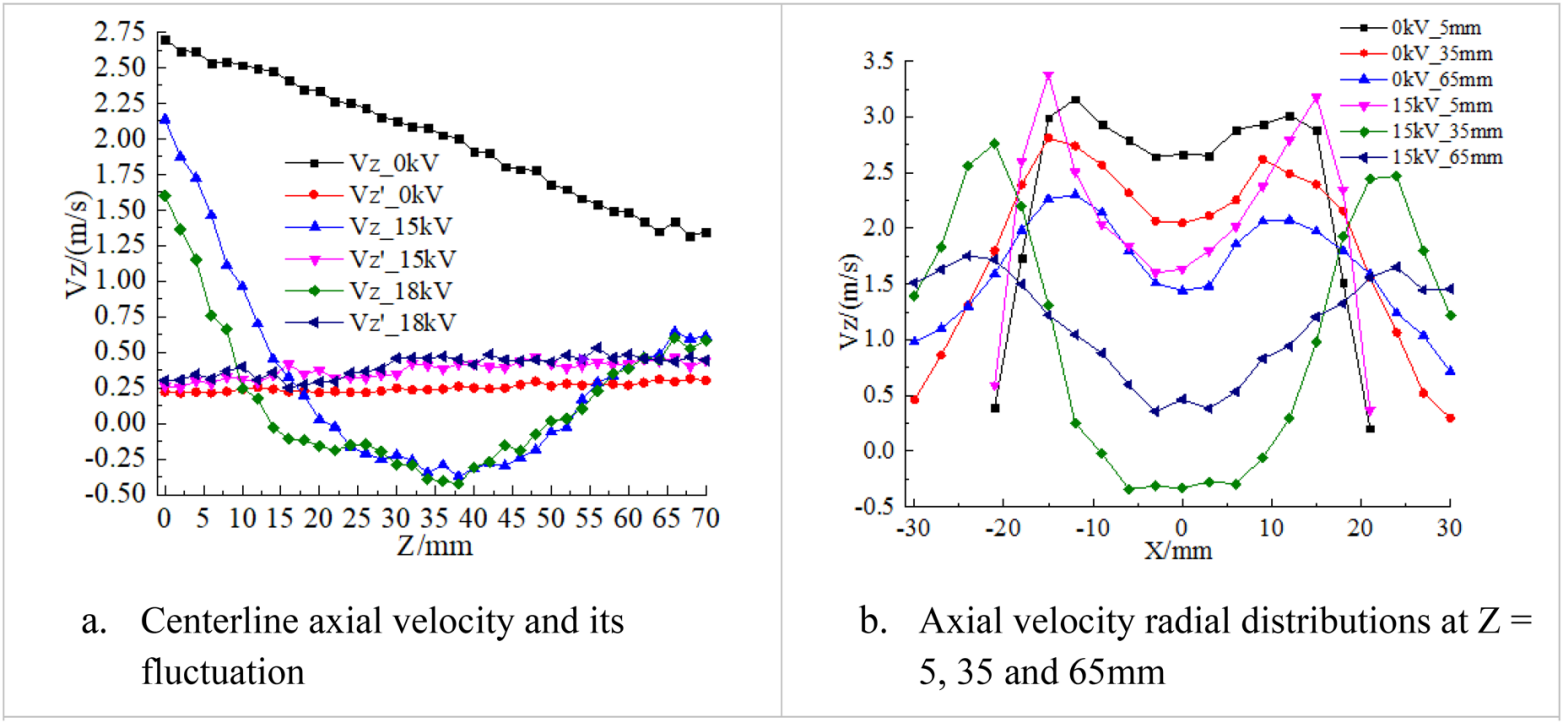
Axial velocity profiles of the non-reacting flows in the centerline and radial direction.

To obtain further insights into the CRZ, centerline axial velocity profiles are measured with applied voltages of 15 kV and 18 kV as shown in Fig. 4a. With the plasma actuation switched off, the centerline axial velocity decreases almost linearly from 2.7 to 1.35 m/s from 0 to 70 mm downstream and the root mean square (RMS) of its fluctuation increases linearly from 0.22 to 0.3 m/s, associated with the downstream flow development. The increase of RMS of velocity fluctuation indicates that the swirling flow becomes more unstable downstream. With the plasma actuation on, the axial velocity decay rate increases remarkably and a CRZ is observed, indicated by the negative values of the axial velocity. It was found that the position of the upstream stagnation point was mainly determined by the strength of the plasma actuation. The upstream stagnation point of the CRZ is at Z = 20 mm and the downstream stagnation point is at Z = 50 mm with 15 kV applied voltage. The minimum axial velocity is about − 0.4 m/s at the center of the CRZ (Z = 36 mm). Increasing the voltage to 18 kV moves the upstream stagnation point 6 mm upstream, but the position of the downstream stagnation point and the value of the minimum velocity remain largely unchanged. The steeper velocity gradient results in higher RMS. Figure 4b shows the axial velocity radial profiles at different locations. With the DBD actuation off, velocity values are all positive and the profiles correspond to the downstream flow development. With the plasma actuation, the velocity profile at Z = 35 shows negative values, indicating the existence of the CRZ.

### Reacting flow field measured by LDA

**Figure 5:**
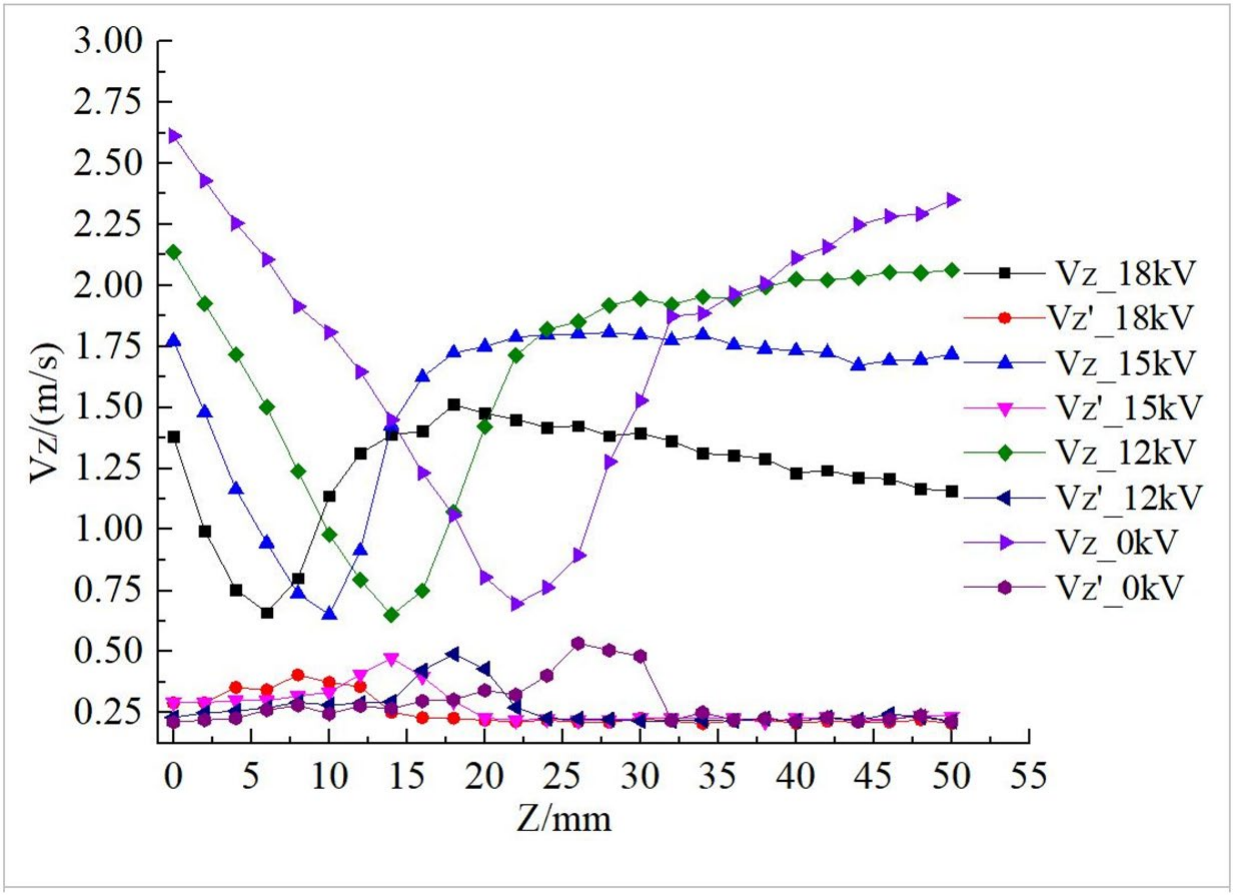
Centerline axial velocity profiles of the reacting flow with the air/CH_4_ volumetric rate at 150/12 L/min respectively.

The reacting flow experiments carried out are similar to a previous study^29^, but with different flow rates (the methane/air are 225/18 L/min and 150/12 L/min respectively). The effect of plasma excitation is stronger in this study because of the lower inflow velocity. The equivalence ratio considered is 0.76. The flame thermal power is about 7.2 kW. The percentage of plasma power source (18 kV) to flame thermal power is about 0.45%. Figure 5 shows the axial velocity and its fluctuation profiles of the reacting flow measured at different voltages. Compared with the non-reacting flow, the presence of the flame increases flow axial stretch rates due to the buoyancy effect and thermal expansion associated with the combustion heat release. Consequently, the velocity field of the reacting flow is very different from that of the non-reacting flow, as indicated by the comparison of the centerline axial velocity profiles shown in Figs. 4a and 5. For the reacting flow, the CRZ disappears as there is no negative velocity along the centerline. Albeit with the existence of the ionic wind generated by the plasma, the effect of flow recirculation is overwhelmed by combustion induced buoyancy and expansion effects. However, the effect of plasma is still significant. The DBD actuation not only shifts the position of the flame front but also increases the mean axial stretch rate represented by the steeper slope of the linear velocity decline at downstream locations^29^. The flame axial leading-edge position shifts depending on the voltage, and the flame lift-off height decreases with increased voltage. This feature can be explored for combustion control. In Fig. 5, the minimum velocity on the axial velocity profile corresponds to the velocity normal to the flame front and provides a means to determine the turbulent flame speed^28^. For those four profiles, the local turbulent flame speed is about 0.65 m/s and the corresponding velocity fluctuation is about 0.33 m/s. Axial velocity downstream the flame front shows a rapid increase due to the combustion heat release accompanied by the increase of its RMS. The RMS reaches its maximum at about 4 mm downstream the flame front edge. In Fig. 5, the RMS values reveal a jump and decay immediately downstream of the flame fronts, exhibiting different peak locations for the cases without plasma actuation (0 kV) and with plasma actuation at different voltages (12 kV, 15 kV, and 18 kV). This is mainly because of the effects of combustion heat release on local turbulence. Immediately downstream of the flame front, the large temperature fluctuations lead to large velocity fluctuations (i.e. large RMS values) which decay quickly outside the flame zone.

**Figure 6:**
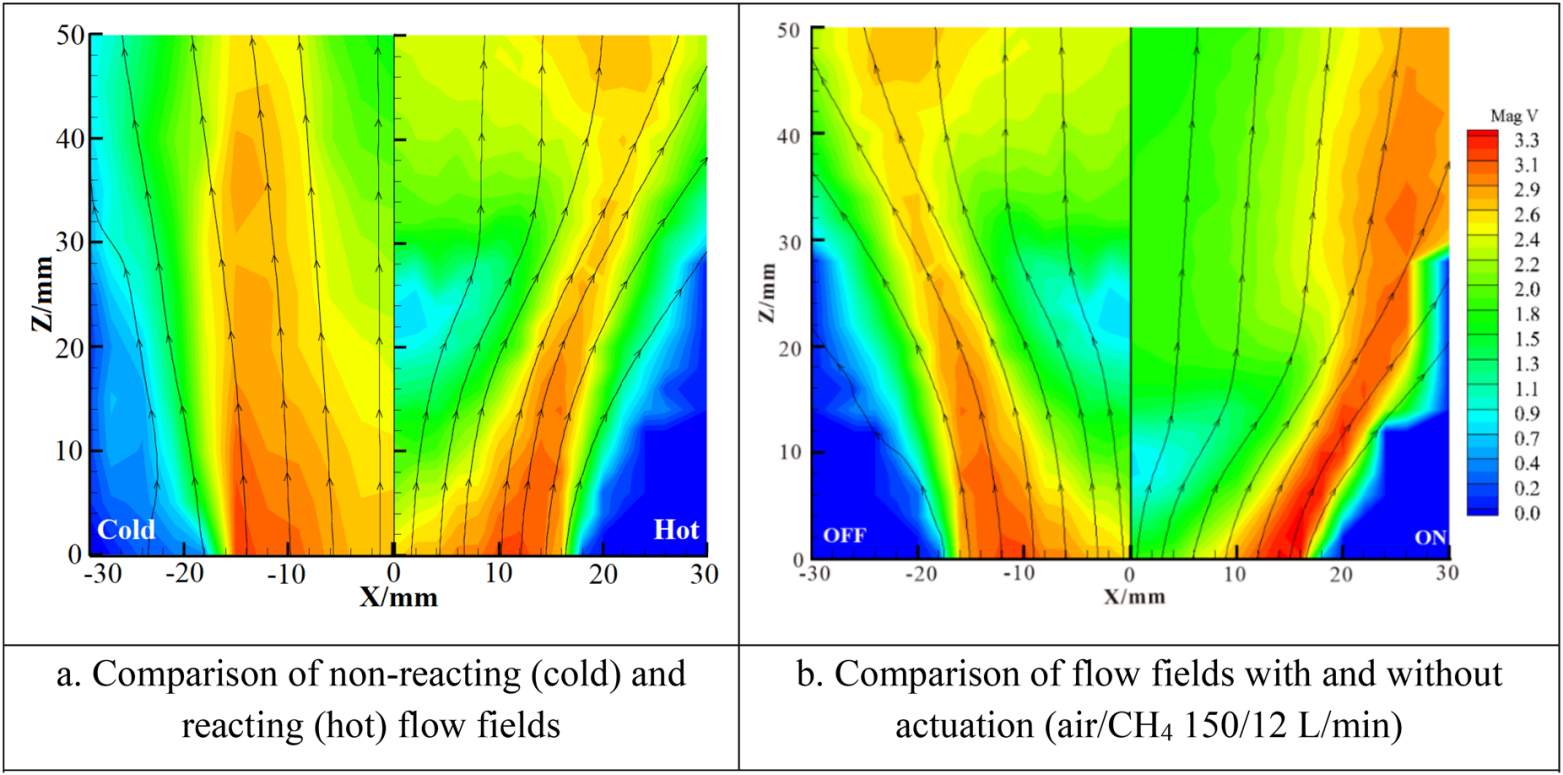
Streamlines and velocity magnitude contours in the XZ plane.

In order to examine the effects of flame and the plasma excitation on velocity field, comparisons of the mean velocity and its fluctuation between two cases without and with combustion are shown in Fig. 6a, while the comparisons between the two cases without and with plasma excitation are shown in Fig. 6b. As shown in Fig. 6, the divergent streamlines near the exit of the injector demonstrate that the reacting flow field expands more in the radial direction than the non-reacting flow in the flow swirling region. The low velocity region near the centerline indicates the position of the front of the flame. As the streamlines develop downstream and through the low velocity region, they bend inwards as a result of the buoyancy effect from the combustion heat release. Further downstream, the streamlines are relatively parallel. The discharge leads to the decrease of V_z_ and increase of V_x_ especially in the central region as shown in Fig. 6b. With the plasma actuation switched on (15 kV voltage), the position and size of the low-speed zone change. The low-speed zone near Z = 18–28 mm moves upstream to Z = 5–12 mm which causes further flow spreading and velocity increase near the rim of the injector exit (X = 10–16 mm). Those changes will greatly affect the position and geometry of the flame.

**Figure 7:**
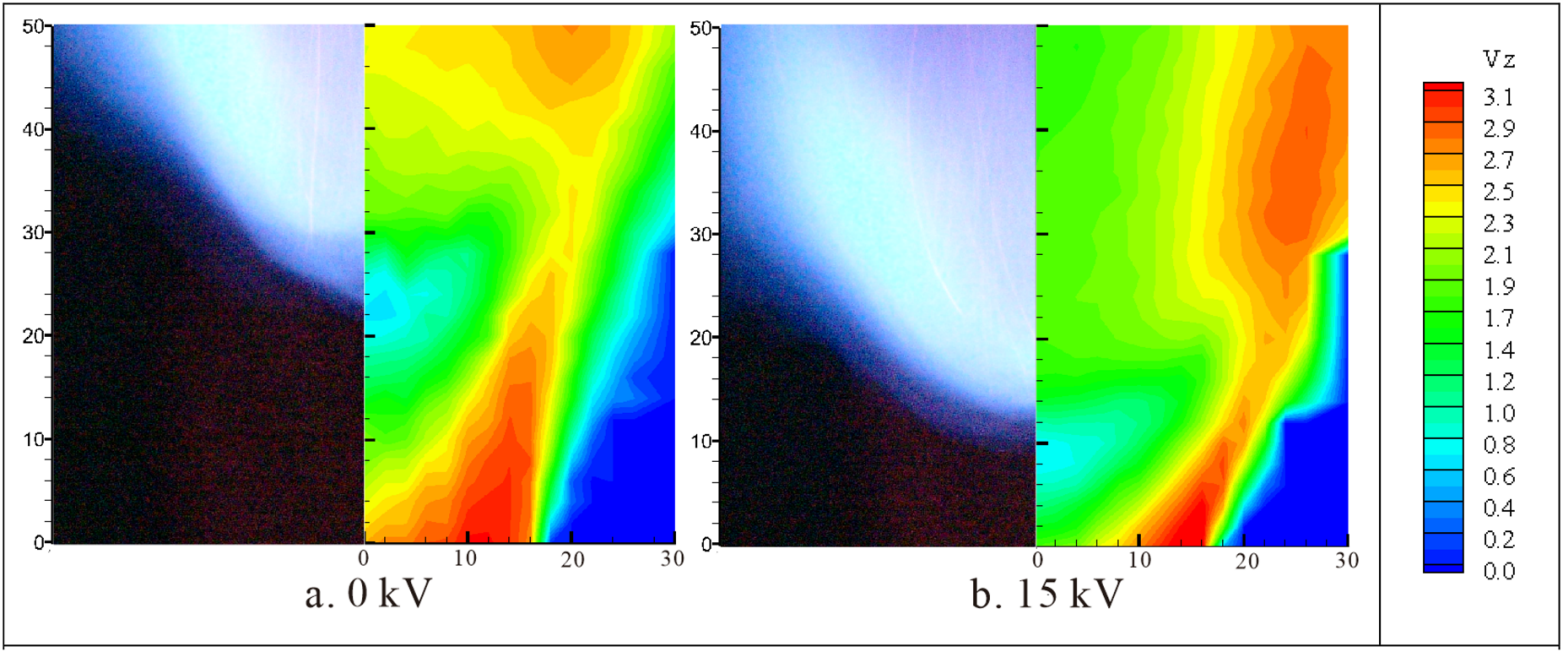
Illustrations of flame image and contours of the axial velocity with the air/CH_4_ volumetric rate at 150/12 L/min respectively.

Figure 7 shows comparison of the flame images with contours of the axial velocity. Both the flame lift-off height and the shape of the flame are sensitive to the plasma actuation. With the actuation of 15 kV, the flame moves upstream and its expansion angle also increases. The change of the flame front position has a significant influence on the mean velocity and its fluctuation.

**Figure 8:**
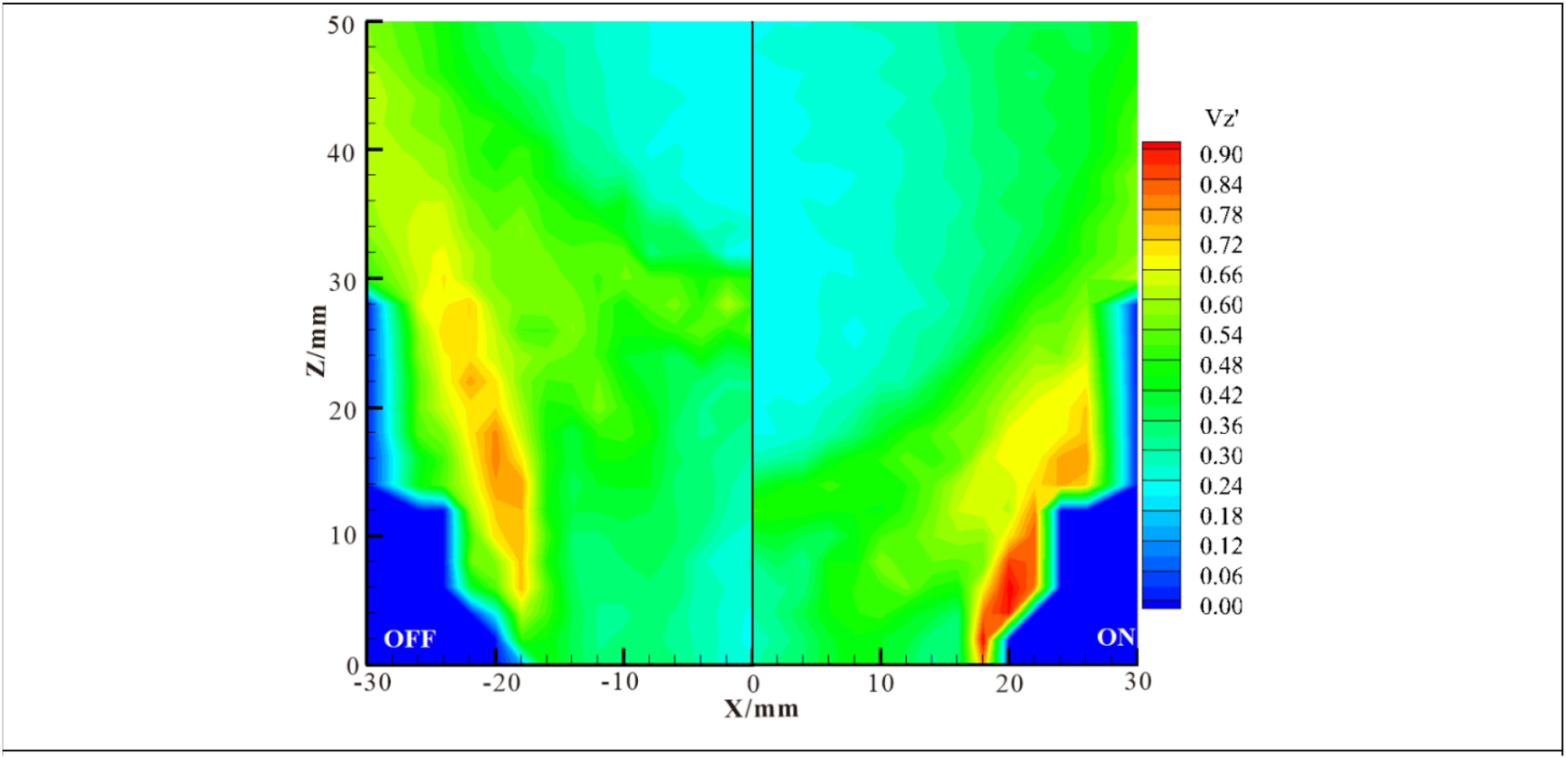
Contours of the axial velocity RMS with the DBD actuation switched off and switched on (15 kV).

Figure 8 shows contours of the RMS of the axial velocity (V_Z_′) with the DBD actuation switched off and on. The contours reveal that the peak values of V_Z_′ are located in the shear layer generated between the swirling jet flow and entrained ambient air. In the axial region, the value of Vz′ is small apart from the flame front. The plasma actuation not only changes the distribution of Vz′ but also increases the Vz′ value above the rim of the injector. The actuation influence on Vz′ is confined near the nozzle rim and does not penetrate into the axial region because the discharge is clinging to the inner wall of the nozzle.

**Figure 9:**
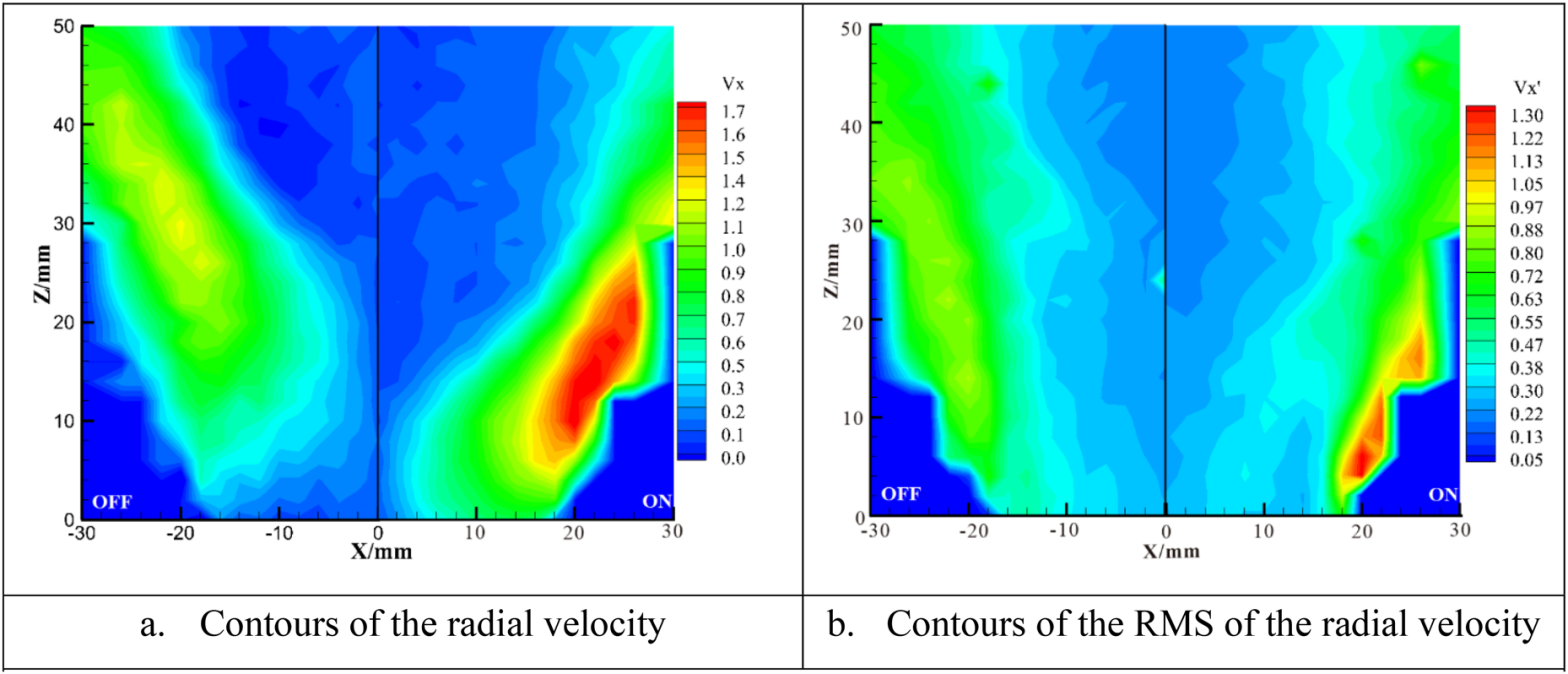
Contours of the radial velocity and its RMS with the DBD actuation switched off and switched on (15 kV).

Figure 9 shows contours of radial velocity with the DBD actuation switched off and on. The value of radial velocity connects with the expansion of the flow field and the radial velocity with high values are located around the flame. In the downstream of the flame front, because of the heat release from combustion, the value of V_X_ is smaller. The plasma actuation draws the flame towards the rim of the injector, affecting the reacting flow field. Accordingly, the distribution of V_X_ changes and the maximum value of V_X_ increases from 1.3 to 1.8 m/s representing a 40% increase. The effect of plasma excitation on Vx′ is similar to that of Vz′ as shown in Fig. 9. The plasma actuation increases the maximum value of V_X_ from 0.7 to 1.3 m/s with an increase of 86%. The value of Vx′ in the central region is low.

## Discussion

DBD plasma actuators have been incorporated into the low swirl burner to enhance the swirl in the non-reacting and reacting flow fields. As a non-intrusive measure, the plasma actuation is effective in flow and combustion control with very small power consumption. The actuation induced CRZ is observed in our burner configuration for the first time and its mechanism is revealed by LDA measurement. The position of the upstream stagnation point was determined by the strength of the actuation, but the position of the downstream stagnation point and the value of the minimum velocity remain largely unchanged. The study is focused on the CRZ induced by the plasma excitation, which is evident in the non-reacting case while the recirculation is significantly weakened in the reacting case because of the combustion heat release. However, plasma excitation still plays a very significant role in the flame dynamics by affecting the fluid dynamic characteristics of the reacting case. In the reacting flow fields, the DBD plasma generates not only an electrical effect but also active species, which affects the flame dynamic behavior including flame position and geometry. The influence of the discharge on the flame lift-off height is observed. An actuation of 18 kV reduces the lift-off height from 24 to 8 mm. Besides, the flow axial stretch rate increases with the plasma actuation. The linear relation between the DBD voltage and the flame lift-off height is demonstrated. For the flow field with the DBD actuation, its features are essentially the same as those of the traditional LSI. Therefore, using the DBD actuation, the swirl can be controlled effectively while the advantages of the LSI can also be retained. The plasma actuation is effective in both the non-reacting and reacting cases, indicating that its effect is mainly aerodynamic. The significant changes in fluid dynamic characteristics demonstrate the effectiveness of plasma actuation in flow and combustion control. The results clearly showed that swirl enhancement in jet flames by the plasma swirler is feasible, flexible and effective and the mechanism of the combustion control by the plasma swirler is mainly through the aerodynamic effect.
